# Supplementary material for: How Do Joint Kinematics and Kinetics Change When Walking Overground with Added Mass on the Lower Body?
Source: Sensors (Basel). 2022 Nov 25;22(23):9177. doi: 10.3390/s22239177 (PMC9738556; doi:10.3390/s22239177)
Supplement: Supplementary file 1 [file sensors-22-09177-s001.zip › sensors-2036491-supplementary.pdf]

Table S1. Participant demographics.

Gender	Number	Age (years)	Body mass (kg)	Body height (m)	BMI (kg / m^2)
Female	10	20.4 ± 1.3	63.2 ± 7.4	1.74 ± 0.07	20.9 ± 2.0
Male	9	25.6 ± 3.4	77.1 ± 13.3	1.78 ± 0.09	24.2 ± 3.6
All	19	22.8 ± 3.6	69.8 ± 12.5	1.76 ± 0.08	22.5 ± 3.3

Table S2. Location and point of application of the 43 markers used during motion capture.

Marker location	Point of application	Total quantity
Acromion (Left & Right)	Clothes	2
Sternum	Clothes	1
Anterior Superior Iliac Spine (Left & Right)	Added mass belt	2
Sacrum	Added mass belt	1
Mid Anterior Superior Iliac Spine (for tracking only)	Added mass belt	1
4-Marker Thigh Plate (Left & Right)	Coban wrap	8
Femur Lateral Epicondyle (Left & Right)	Skin	2
Femur Medial Epicondyle (Left & Right)	Skin	2
4-Marker Shank Plate (Left & Right)	Coban wrap	8
Ankle Lateral Malleolus (Left & Right)	Skin/Clothes	2
Ankle Medial Malleolus (Left & Right)	Skin/Clothes	2
Foot (Left & Right)	Shoes	12

Table S3. Highest Pearson Correlation coefficients.

Dependent variable	Pearson Correlation coefficient
Stride Length	0.551
HE Moment	0.485
Gait Time	0.397
Stance Time	0.394
Stride Rate	0.393
Cadence	0.393
Single Support Time	0.387
Gait Speed	0.344
Double Support Time II	0.329
KE Moment	0.326
Swing Time	0.323
HF2	0.303
HF Moment	0.300
KF2	0.296
AP1	0.291
Knee RoM	0.267
AD	0.258
AP2	0.224
AP Moment	0.203
KF1	0.182
Double Support Time I	0.174
HF1	0.173
Hip RoM	0.172
KF3	0.163
Ankle RoM	0.162
HE	0.151
KF Moment	0.132
KE	0.095

The coefficients shown here are the highest magnitudes across all independent variables. Independent variables: Age (years), Body mass (kg), Body height (m), and BMI (kg/m^2). Gender is assessed as a "Between Factor" in Repeated Measures ANOVA, all $p > 0.050$ ($\alpha = .050$).

Table S4. Mean and Standard Deviation values of sagittal-plane range of motion (RoM) in degrees.

Amount	Location	Hip RoM	Knee RoM	Ankle RoM
Baseline	-	33.605 (4.983)	68.172 (4.252)	47.750 (4.497)
Low	Pelvis	33.630 (5.347)	69.009 (3.938)	48.427 (4.959)
Low	Thigh	33.268 (4.771)	68.305 (3.712)	47.561 (4.949)
Low	Shank	32.558 (5.689)	68.093 (4.288)	**48.399 (4.742)**
High	Pelvis	32.918 (5.643)	68.575 (4.147)	47.988 (5.722)
High	Thigh	33.090 (5.179)	66.686 (4.683)	46.667 (4.616)
High	Shank	32.220 (5.485)	67.133 (4.554)	**48.551 (4.801)**

Bold items are significantly different from the Baseline condition (both $p < 0.020$, $\alpha = .050$).

Table S5. Mean and Standard Deviation values of the spatiotemporal metrics.

Amount	Location	Stride Length (m)	Stride Rate (Hz)	Cadence (steps/min)	Gait Speed (m/s)	Stance Time (s)	Swing Time (s)	Double Support Time I (s)	Double Support Time II (s)	Single Support Time (s)
Baseline	-	1.444 (0.104)	0.890 (0.061)	106.783 (7.305)	1.286 (0.139)	0.698 (0.053)	0.431 (0.028)	0.149 (0.019)	0.139 (0.021)	0.411 (0.026)
Low	Pelvis	1.448 (0.106)	0.891 (0.061)	106.937 (7.293)	1.292 (0.142)	0.697 (0.052)	0.431 (0.030)	0.150 (0.017)	0.140 (0.021)	0.406 (0.028)
Low	Thigh	1.453 (0.109)	0.886 (0.054)	106.351 (6.447)	1.288 (0.137)	0.699 (0.049)	0.434 (0.026)	0.149 (0.018)	0.139 (0.022)	0.411 (0.025)
Low	Shank	1.448 (0.114)	0.887 (0.060)	106.485 (7.222)	1.286 (0.144)	0.699 (0.053)	0.433 (0.031)	0.146 (0.018)	0.136 (0.021)	0.417 (0.027)
High	Pelvis	1.435 (0.117)	0.881 (0.059)	105.724 (7.112)	**1.265 (0.143)**	0.706 (0.053)	0.434 (0.031)	**0.154 (0.017)**	**0.144 (0.021)**	0.407 (0.025)
High	Thigh	1.446 (0.114)	0.881 (0.052)	105.731 (6.235)	1.275 (0.132)	0.701 (0.046)	**0.438 (0.026)**	0.149 (0.015)	0.138 (0.019)	0.414 (0.026)
High	Shank	1.445 (0.117)	0.882 (0.061)	105.843 (7.370)	1.276 (0.152)	0.700 (0.053)	**0.439 (0.033)**	0.145 (0.018)	**0.134 (0.023)**	**0.421 (0.028)**

Bold items are significantly different from the Baseline condition (all $p < 0.050$, $\alpha = .050$).

Table S6. P-Value (Power) for analyzed metrics with two-way, repeated measures ANOVA.

Metric	Mass Location factor	Mass Amount factor
Stride Length	0.008 (0.804)	0.008 (0.804)
Stride Rate	0.000 (0.997)	0.000 (0.997)
Cadence	0.000 (0.997)	0.000 (0.997)
Gait Speed	0.000 (0.995)	0.000 (0.995)
Stance Time	0.005 (0.844)	0.005 (0.844)
Swing Time	0.000 (0.998)	0.000 (0.998)
Double Support Time I	0.188 (0.354)	0.188 (0.354)
Double Support Time II	0.848 (0.076)	0.848 (0.076)
Single Support Time	0.016 (0.732)	0.016 (0.732)
Gait Time	0.000 (0.996)	0.000 (0.996)
AP1	0.000 (0.999)	0.000 (0.999)
AD	0.000 (1.000)	0.000 (1.000)
AP2	0.000 (1.000)	0.000 (1.000)
Ankle RoM	0.000 (0.995)	0.000 (0.995)
KF1	0.022 (0.697)	0.022 (0.697)
KF2	0.001 (0.930)	0.001 (0.930)
KE	0.000 (0.951)	0.000 (0.951)
KF3	0.000 (0.999)	0.000 (0.999)
Knee RoM	0.000 (1.000)	0.000 (1.000)
HF1	0.000 (0.999)	0.000 (0.999)
HE	0.000 (0.975)	0.000 (0.975)
HF2	0.007 (0.808)	0.007 (0.808)
Hip RoM	0.023 (0.690)	0.023 (0.690)
AP Moment	0.000 (1.000)	0.000 (1.000)
KE Moment	0.000 (1.000)	0.000 (1.000)
KF Moment	0.044 (0.604)	0.044 (0.604)
HF Moment	0.000 (1.000)	0.000 (1.000)
HE Moment	0.005 (0.836)	0.005 (0.836)

Values are extracted and rounded through NCSS (α = .050).

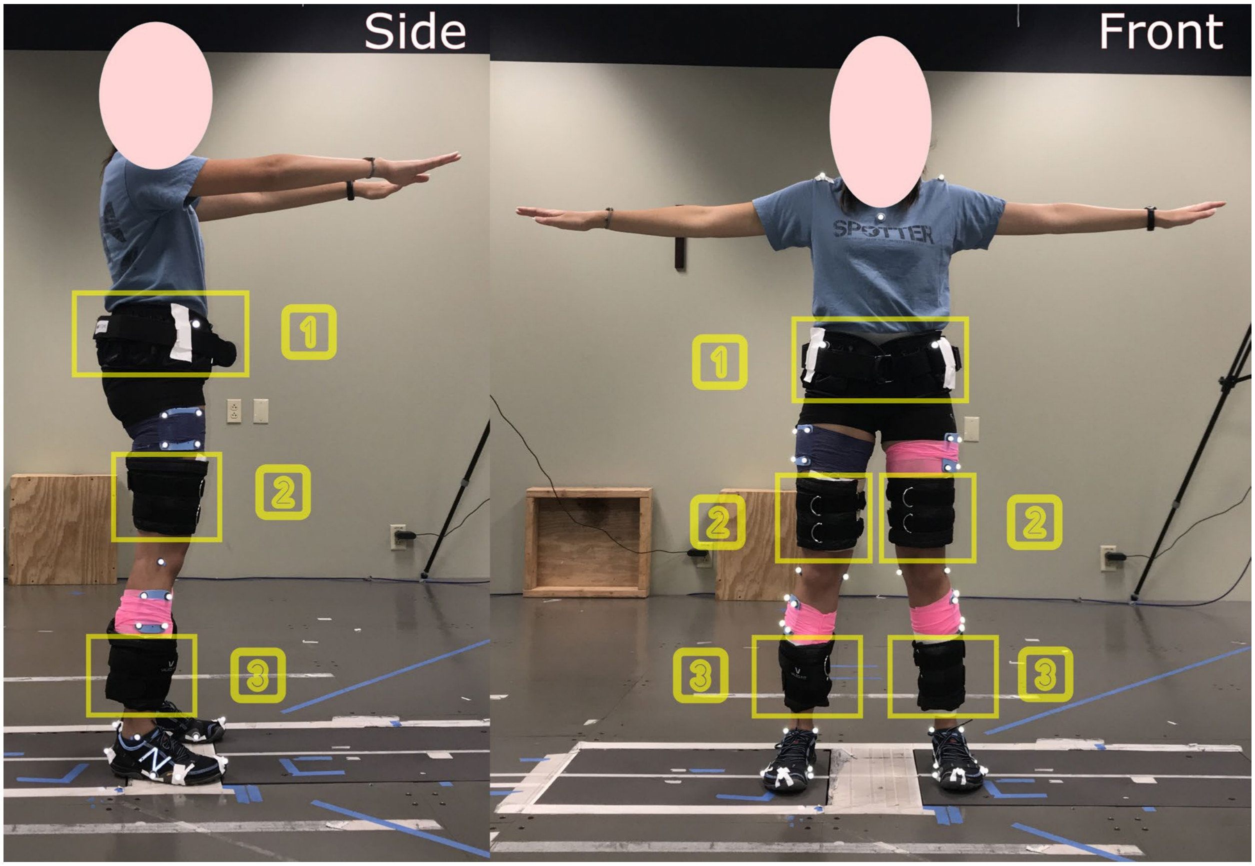

Figure S1. Front and side views showing the placement of markers and belts containing the added masses. Sacrum marker is not shown here. Brackets show the location of the added masses. Bracket labeled “1”: Added mass at the Pelvis location with an amount of 0/+8/+16 lb; Brackets labeled “2”: Added mass at the Thigh location each with an amount of 0/+2/+4 lb; Brackets labeled “3”: Added mass at the Shank location each with an amount of 0/+2/+4 lb.

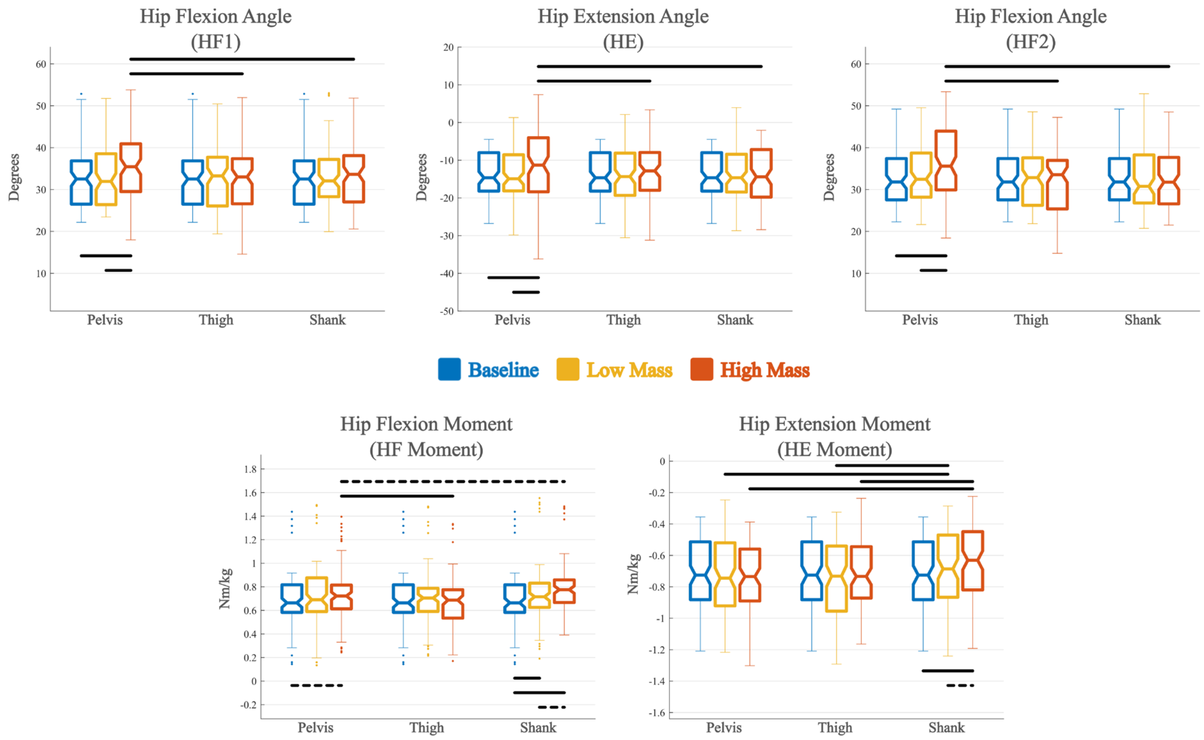

Figure S2. Box plots of peak hip joint kinematic and kinetics at different added mass locations. Each pair of conditions significantly different from each other is connected by a horizontal bar ($\alpha = 0.050$). Type of the bar indicates the significance level: dashed for $0.001 \leq p < 0.050$ and solid for $p < 0.001$.

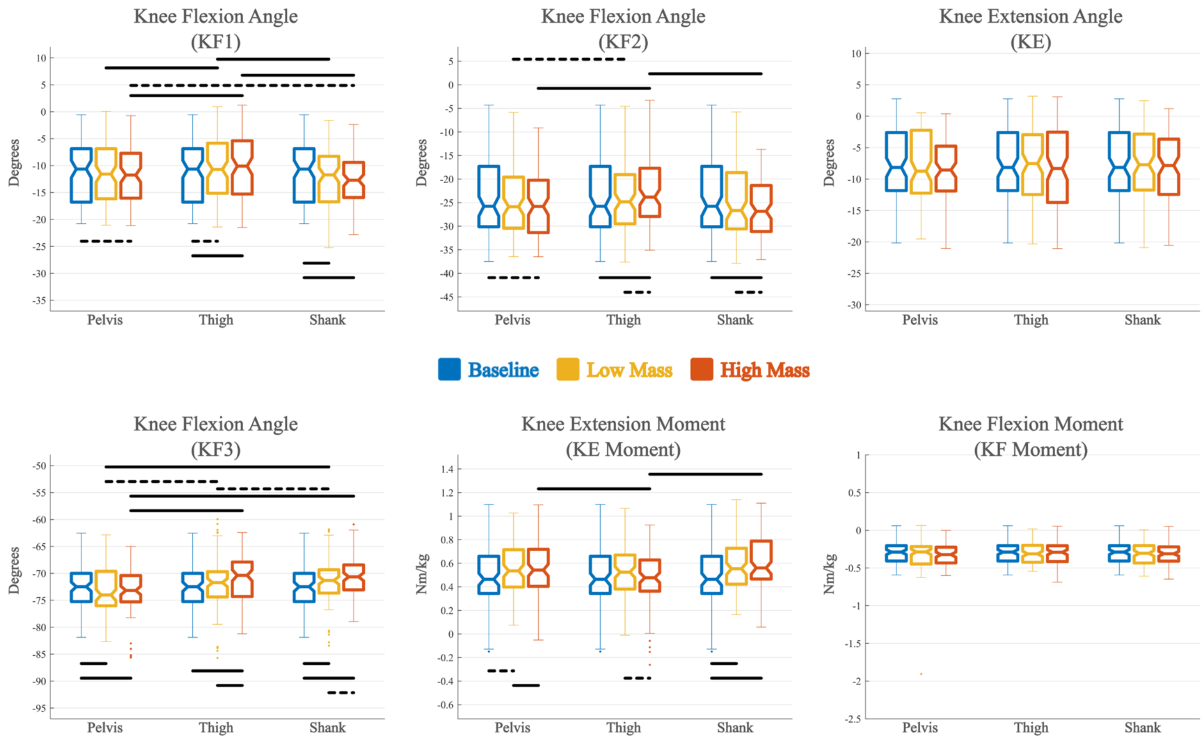

Figure S3. Box plots of peak knee joint kinematic and kinetics at different added mass locations. Each pair of conditions significantly different from each other is connected by a horizontal bar ($\alpha = 0.050$). Type of the bar indicates the significance level: dashed for $0.001 \leq p < 0.050$ and solid for $p < 0\ .001$.

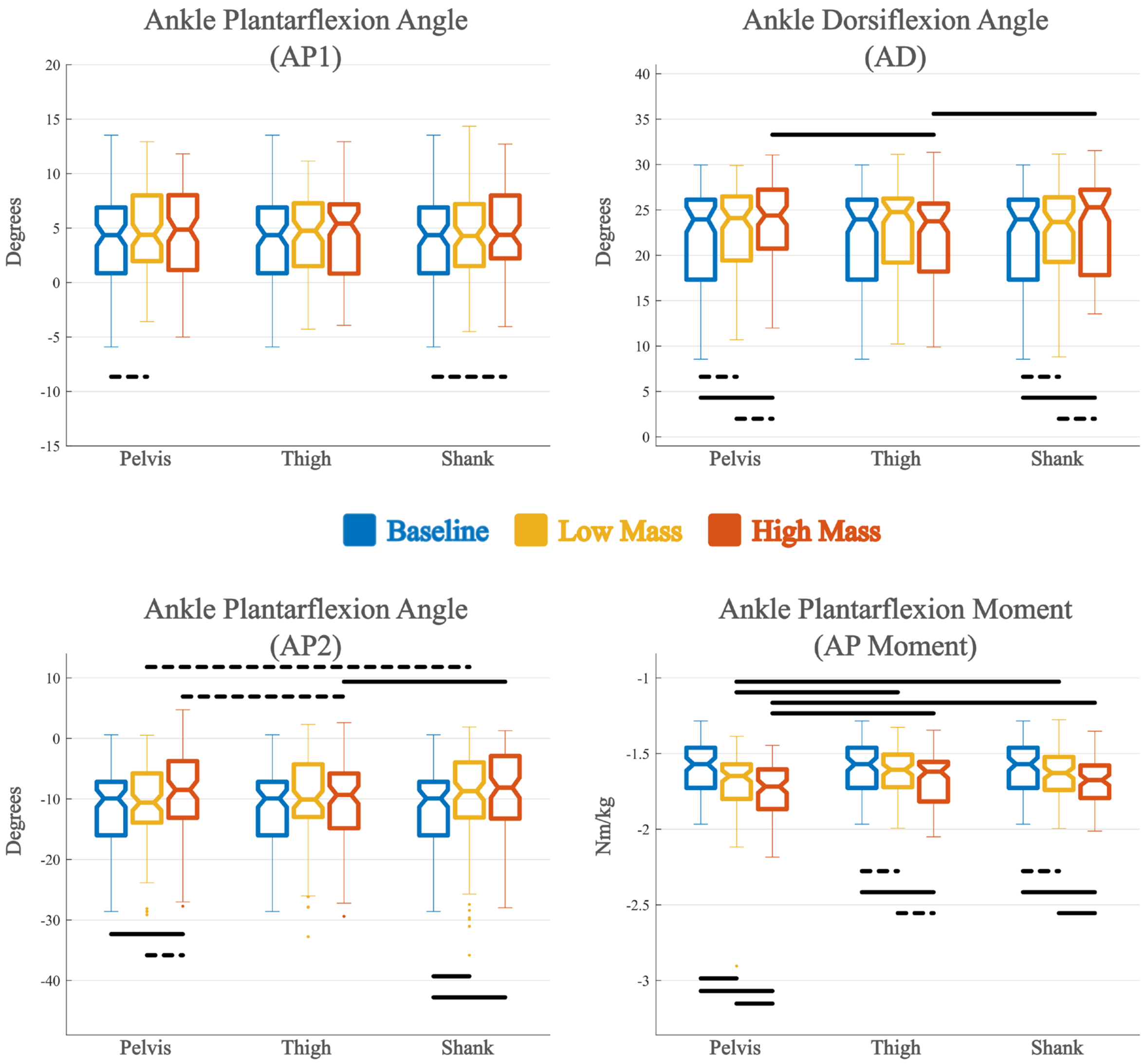

Figure S4. Box plots of peak ankle joint kinematic and kinetics at different added mass locations. Each pair of conditions significantly different from each other is connected by a horizontal bar ($\alpha = 0.050$). Type of the bar indicates the significance level: dashed for $0.001 \leq p < 0.050$ and solid for $p < 0.001$.

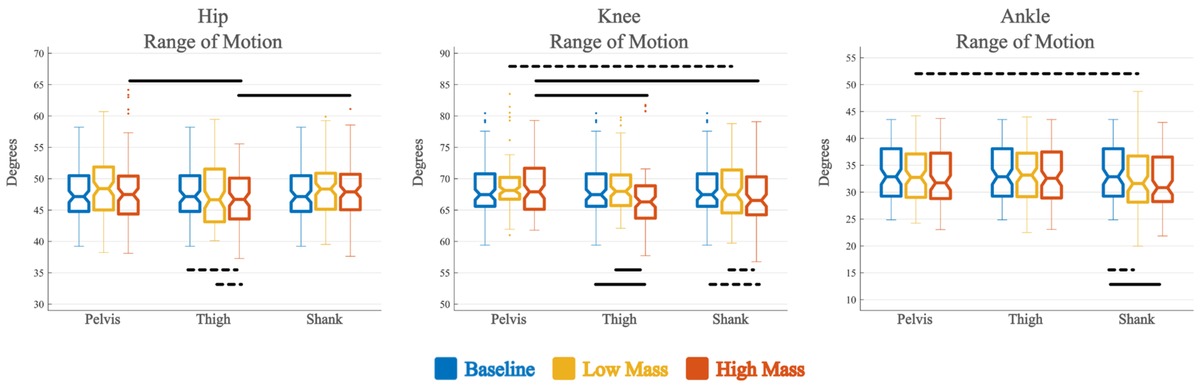

Figure S5. Box plots of range of motion at different added mass locations. Each pair of conditions significantly different from each other is connected by a horizontal bar ($\alpha = 0.050$). Type of the bar indicates the significance level: dashed for $0.001 \leq p < 0.050$ and solid for $p < 0.001$.

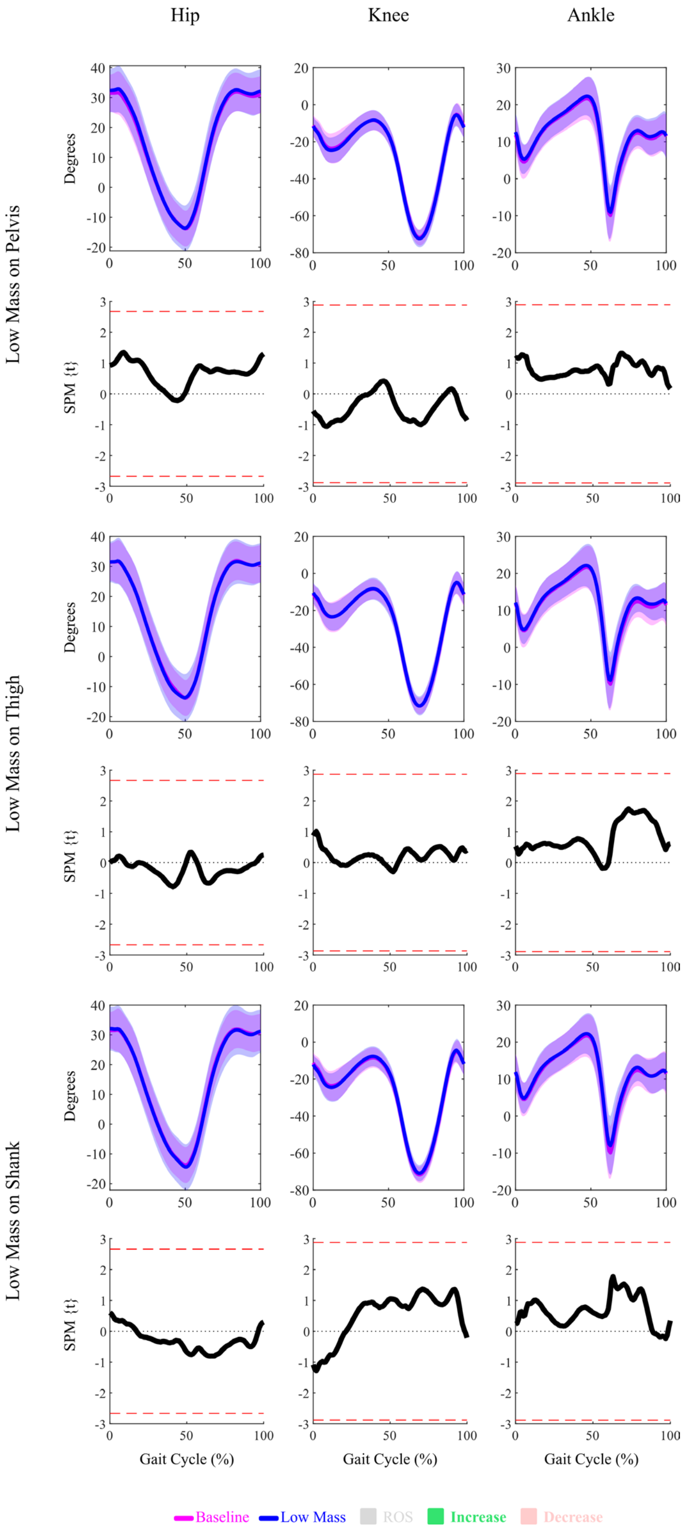

Figure S6. Mean and Standard Deviation plots of the joint kinematics under both the Low Mass conditions and the Baseline condition, and SPM{t} plots demonstrating the significance (Region of Significance, or ROS) of the comparisons between the Low Mass and the Baseline conditions. Upon significance, directions of changes are highlighted in color.

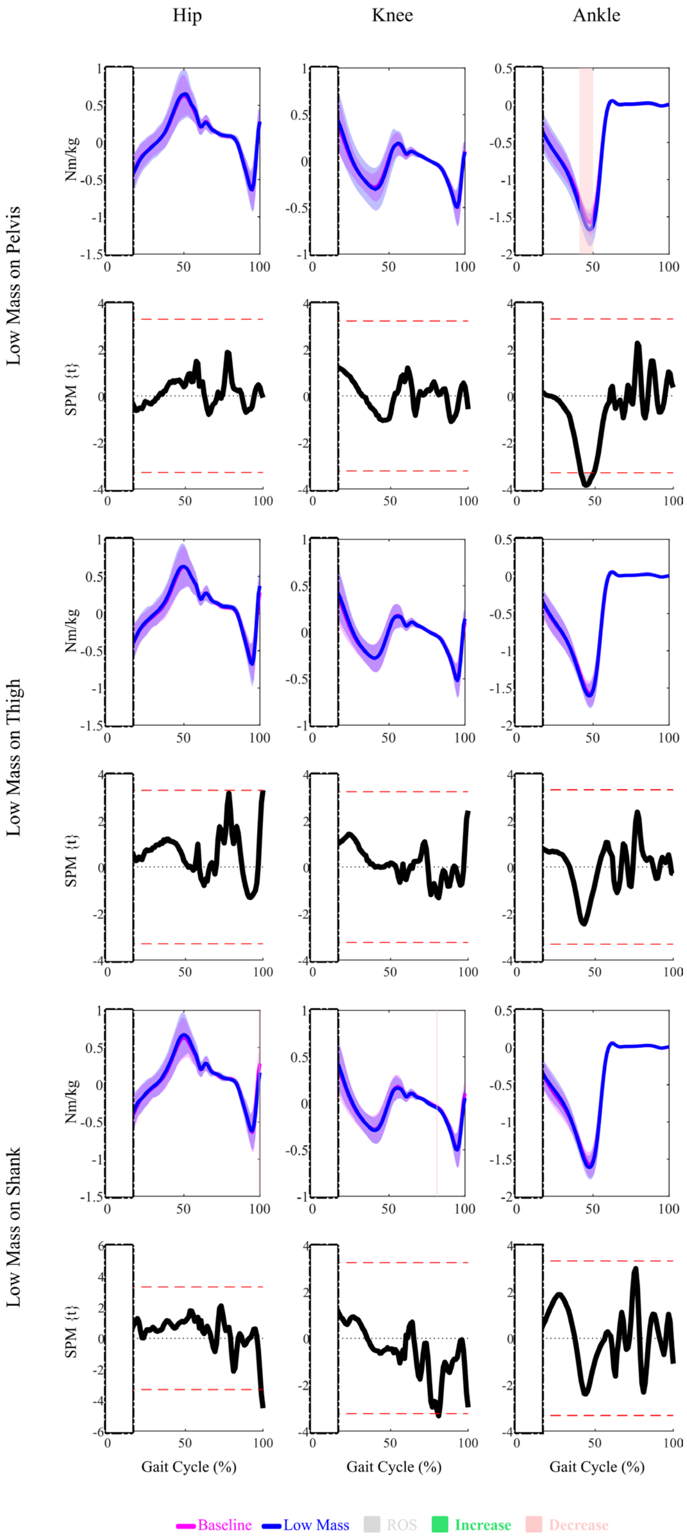

Figure S7. Mean and Standard Deviation plots of the joint kinetics under both the Low Mass conditions and the Baseline condition, and SPM{t} plots demonstrating the significance (Region of Significance, or ROS) of the comparisons between the Low Mass and the Baseline conditions. Upon significance, directions of changes are highlighted in color. Rectangles with dash-dotted edges are used to mask the early stance joint kinetics for lack of necessary ground reaction force data.
